# Endoscopic stapes surgery for otosclerosis: clinical, radiological, and audiological outcomes in a retrospective series of 40 patients

**DOI:** 10.1097/MS9.0000000000005239

**Published:** 2026-06-30

**Authors:** Saad Bouchlarhem, Achraf Amine Sbai, Drissia Benfadil, Azeddine Lachkar

**Affiliations:** a Faculty of Medicine and Pharmacy, Mohammed First University, Oujda, Morocco; b Department of Otorhinolaryngology, Mohammed VI University Hospital, Mohammed I University, Oujda, Morocco; c Laboratory of Oto-Neuro-Ophthalmology (LORNO), Faculty of Medicine and Pharmacy, Mohammed First University, Oujda, Morocco; d Faculty of Medicine and Pharmacy, Mohammed First University, LAMCESM, Oujda, Morocco

**Keywords:** air–bone gap, conductive hearing loss, endoscopic ear surgery, otosclerosis, stapes surgery

## Abstract

**Background:**

Otosclerosis is a primary osteodystrophy of the otic capsule, characterized by abnormal bone remodeling, leading to stapes fixation and progressive conductive or mixed hearing loss. Although microscopic stapes surgery remains the historical standard, endoscopic ear surgery has gained increasing interest due to improved visualization of middle ear anatomy and its minimally invasive transcanal approach. This study aimed to evaluate the clinical, radiological, and audiological outcomes of exclusive endoscopic stapes surgery in a retrospective cohort of patients with otosclerosis.

**Methods:**

We conducted a retrospective observational cohort study including 40 patients who underwent exclusive transcanal endoscopic stapedotomy for otosclerosis at the Department of Otorhinolaryngology, Mohammed VI University Hospital, Oujda, between January 2023 and December 2025. Postoperative pure-tone audiometry was systematically performed 3 months after surgery (±2 weeks). Patients without audiometric evaluation at this predefined endpoint were excluded. Air-conduction (AC) and bone-conduction (BC) pure-tone averages (0.5–1–2–4 kHz) were recorded pre- and postoperatively. The air–bone gap (ABG) was calculated as the difference between AC-PTA and BC-PTA. Pre- and postoperative outcomes were compared using paired statistical analysis.

**Results:**

Forty patients (mean age 34 years) were included. The mean preoperative ABG was 32 ± 6 dB and decreased to 9 ± 4 dB postoperatively, corresponding to a mean improvement (ΔABG) of 23 dB (*P* < 0.001). The majority of patients achieved functional ABG closure (≤10 dB). Mean AC-PTA improved significantly, while BC-PTA remained stable, with no cases of postoperative sensorineural hearing loss defined as BC deterioration ≥10 dB. Endoscopic stapedotomy was successfully completed in all cases. Complications were limited to transient minor events, with no facial nerve dysfunction or persistent vertigo.

**Conclusion:**

Exclusive transcanal endoscopic stapes surgery is a safe, reproducible, and effective technique for the management of otosclerosis. The endoscopic approach provides optimal visualization of middle ear anatomy and yields favorable functional outcomes with low morbidity, supporting its role as a reliable alternative to conventional microscopic techniques in experienced centers.

## Introduction

Otosclerosis is a primary localized disorder of bone remodeling of the otic capsule, characterized by abnormal osteodystrophy leading to progressive fixation of the stapes footplate. This condition disrupts sound transmission to the inner ear and typically manifests as progressive conductive hearing loss, which may evolve into mixed or sensorineural hearing loss (SNHL) in cases of cochlear involvement. Otosclerosis predominantly affects young and middle-aged adults and remains one of the most common cau/ses of conductive hearing loss in patients with an intact tympanic membrane. Diagnosis is based on clinical presentation, audiometric demonstration of an air–bone gap (ABG), and high-resolution temporal bone computed tomography (CT), which may reveal characteristic hypodense foci at the fissula ante fenestram^[^1,2^]^.

Surgical management remains the treatment of choice for patients with functionally significant hearing loss. Conventional microscopic stapes surgery – particularly stapedotomy – has long been considered the gold standard, providing predictable and favorable audiological outcomes^[^3^]^. However, the microscopic approach may be limited by a restricted line of sight, especially in patients with anatomical constraints such as a narrow external auditory canal, overhanging scutum, or a prominent facial nerve^[^4^]^. These limitations have stimulated interest in alternative visualization techniques aimed at improving exposure and surgical precision.

Endoscopic ear surgery has emerged over the past decade as a minimally invasive alternative in otologic procedures, including stapedotomy. The endoscopic approach offers a wide-angle, high-definition view of the middle ear, allowing improved visualization of the oval window niche, stapes suprastructure, facial nerve, and ossicular chain without extensive bone removal. This enhanced exposure may facilitate more precise prosthesis placement while preserving surrounding anatomical structures. Despite growing adoption, evidence regarding the effectiveness, safety, and reproducibility of exclusive endoscopic stapedotomy remains limited, particularly in homogeneous case series with standardized follow-up protocols^[^5–7^]^. The present study aimed to evaluate the clinical characteristics, radiological patterns, intraoperative findings, and audiological outcomes of exclusive transcanal endoscopic stapedotomy in a retrospective cohort of 40 patients.

## Patients and methods

### Study design and setting

A retrospective observational cohort study was conducted in the Department of Otorhinolaryngology at Mohammed VI University Hospital Center, Oujda. Institutional surgical, audiological, and radiological databases were reviewed to identify all patients who underwent exclusive transcanal endoscopic stapes surgery for otosclerosis between January 2023 and December 2025.

The primary objective was to evaluate clinical characteristics, radiological patterns, intraoperative findings, and audiological outcomes following an endoscopic approach.

### Patient selection

Patients were eligible if they had confirmed or highly suspected otosclerosis and were managed surgically using a purely endoscopic transcanal approach.

**Confirmed otosclerosis** was defined by:

- Progressive conductive or mixed hearing loss.
- ABG on pure-tone audiometry.
- Compatible high-resolution temporal bone CT findings (fenestral or cochlear lesions according to the Veillon classification).
- Intraoperative confirmation of stapes fixation.

**Highly suspected otosclerosis** was defined by:

- Progressive conductive hearing loss with significant ABG.
- Normal tympanic membrane.
- Absence of alternative middle ear pathology.
- Typical clinical presentation (including possible paracusis of Willis).
- CT findings that were normal or equivocal (Veillon Type 0).
- Intraoperative confirmation of stapes fixation.

Patients were excluded if they had:

- Incomplete or non-exploitable medical records.
- Absence of postoperative audiometric evaluation at the predefined endpoint.
- Non-endoscopic surgical management.
- Alternative diagnoses identified intraoperatively.
- Loss to follow-up before postoperative assessment.

During the study period, six additional patients were excluded due to incomplete follow-up or missing audiometric data.

HIGHLIGHTS

- Endoscopic stapes surgery is a safe and effective surgical option for the management of otosclerosis.
- Exclusive endoscopic transcanal stapes surgery provides excellent visualization of middle ear anatomy, even in anatomically challenging cases.
- Significant postoperative hearing improvement was achieved, with satisfactory air–bone gap closure in the majority of patients.
- The endoscopic approach demonstrated a low complication rate, with no major adverse events observed.
- Endoscopic stapes surgery represents a reliable alternative to conventional microscopic techniques in appropriately selected patients.

Baseline demographic and clinical characteristics are summarized in Table 1.

**Table 1:** Demographic and clinical characteristics of the study population (*n* = 40).

Variable	Value
Mean age (years)	34 (range: 26–50)
Male sex	21 (52.5%)
Female sex	19 (47.5%)
Mean duration of symptoms (years)	7 (range: 2–15)
Progressive hearing loss	40 (100%)
Tinnitus	24 (60%)
Vestibular symptoms	5 (12.5%)
Paracusis of Willis	3 (7.5%)
Unilateral hearing loss	28 (70%)
Bilateral hearing loss	12 (30%)
Normal tympanic membrane	40 (100%)

### Audiological evaluation

Pure-tone audiometry was performed preoperatively and systematically at 3 months postoperatively (±2 weeks). The minimum follow-up required for inclusion was 3 months.

Air-conduction (AC) and bone-conduction (BC) thresholds were measured at 0.5, 1, 2, and 4 kHz. AC pure-tone average (AC-PTA) and BC pure-tone average (BC-PTA) were calculated as the arithmetic mean of these four frequencies.

The ABG was defined as:

ABG = AC-PTA − BC-PTA

The primary audiological endpoint was postoperative ABG and the rate of functional ABG closure. Functional success was defined as an ABG ≤10 dB at the postoperative endpoint, in accordance with widely accepted reporting standards in stapes surgery literature^[^8^]^.

Hearing loss was classified as conductive or mixed, and staged according to the Juers and Shambaugh classification.

Postoperative SNHL was defined as a deterioration in BC-PTA of ≥10 dB compared to preoperative values. The incidence of BC deterioration was systematically assessed.

Preoperative audiological data are presented in Table 2. Postoperative audiological outcomes are summarized in Table 3.

**Table 2:** Preoperative audiological characteristics.

Variable	Value
Pure conductive hearing loss	22 (55%)
Mixed hearing loss	18 (45%)
Stage I (pure conductive)	22 (55%)
Stage II (early cochlear involvement)	12 (30%)
Stage III (significant cochlear involvement)	6 (15%)
Stage IV	0 (0%)
Mean preoperative ABG (0.5–1–2–4 kHz)	32 ± 6 dB

**Table 3:** Postoperative audiological outcomes.

Variable	Value
Mean postoperative ABG (0.5–1–2–4 kHz)	9 ± 4 dB
Mean ABG improvement	23 dB
ABG ≤ 10 dB	28 (70%)
ABG 11–20 dB	10 (25%)
ABG > 20 dB	2 (5%)
Subjective hearing improvement	38 (95%)
Postoperative SNHL (≥10 dB BC worsening)	0 (0%)

### Radiological assessment

All patients underwent high-resolution temporal bone CT prior to surgery. Imaging findings were classified according to the Veillon classification, allowing a standardized evaluation of fenestral and cochlear involvement.

Radiological findings are summarized in Table 4.

**Table 4:** Radiological findings according to the Veillon classification.

Variable	Value
Veillon type 0	4 (10%)
Veillon type 1A	8 (20%)
Veillon type 1B	11 (27.5%)
Veillon type 2	10 (25%)
Veillon type 3	7 (17.5%)
Veillon type 4	0 (0%)

### Surgical technique

All procedures were performed using an exclusive transcanal endoscopic approach under general anesthesia. Visualization was achieved using a rigid 3-mm 0° endoscope (Karl Storz, Tuttlingen, Germany), with occasional use of a 30° endoscope when required.

A tympanomeatal flap was elevated to expose the middle ear. Limited curettage of the posterior scutum was performed, when necessary, to improve visualization while preserving the chorda tympani nerve whenever feasible.

After confirming stapes fixation, the incudostapedial joint was disarticulated, and the stapedial tendon was sectioned. A stapedotomy was performed using a microperforator to create a small fenestra in the fixed footplate.

A 0.6-mm Teflon piston prosthesis was inserted. The prosthesis length was determined intraoperatively by measuring the distance between the long process of the incus and the footplate. The piston was crimped onto the incus to ensure stable fixation.

The oval window was sealed using a small fragment of connective tissue harvested from the tragus. The tympanomeatal flap was repositioned, and the ear canal was packed with absorbable material.

All procedures were performed by two experienced otologic surgeons trained in endoscopic ear surgery. Intraoperative findings are summarized in Table 5.

**Table 5:** Intraoperative findings.

Variable	Value
Narrow external auditory canal	9 (22.5%)
Overhanging scutum	11 (27.5%)
Prominent facial nerve	4 (10%)
Minor bleeding	3 (7.5%)
Floating footplate	2 (5%)
No intraoperative incident	35 (87.5%)

### Postoperative follow-up

Patients were clinically evaluated during the early postoperative period and underwent standardized audiological assessments at 3 months postoperatively. Early and late complications, including vertigo, dysgeusia, facial nerve dysfunction, perilymphatic fistula, and delayed sensorineural deterioration, were recorded.

Postoperative complications are summarized in Table 6.

**Table 6:** Postoperative complications.

Variable	Value
Transient vertigo	4 (10%)
Dysgeusia	3 (7.5%)
Facial nerve dysfunction	0 (0%)
Perilymphatic fistula	0 (0%)

### Statistical analysis

Quantitative variables were expressed as mean ± standard deviation or range, while qualitative variables were presented as absolute numbers and percentages.

Preoperative and postoperative ABG values were compared using a paired Student’s *t*-test. The mean difference in ABG was calculated, and a 95% confidence interval was estimated. A *P*-value < 0.05 was considered statistically significant.

Statistical analyses were performed using Microsoft Excel (2019).

## Results

During the study period, a total of 40 patients underwent exclusive transcanal endoscopic stapes surgery for otosclerosis and were included in the final analysis. The mean age of the study population was 34 years (range: 26–50 years), with a slight male predominance (male-to-female ratio: 1.1). Progressive hearing loss was the presenting symptom in all patients, while tinnitus was reported in 60% of cases and vestibular symptoms in 12.5%. The mean duration of symptoms prior to surgery was 7 years (range: 2–15 years). Baseline demographic and clinical characteristics are summarized in Table 1.

Preoperative audiological assessment revealed pure conductive hearing loss in 55% of patients (*n* = 22) and mixed hearing loss in 45% (*n* = 18). According to the Juers and Shambaugh audiometric classification, stage I disease was observed in 55% of cases, stage II in 30%, and stage III in 15%, with no patients presenting with stage IV disease. Detailed preoperative audiological characteristics are presented in Table 2.

The mean preoperative ABG, calculated from pure-tone averages at 0.5, 1, 2, and 4 kHz, was 32 ± 6 dB. At the predefined postoperative endpoint, the mean ABG decreased to 9 ± 4 dB, corresponding to a mean ABG improvement of 23 dB. Paired comparison analysis demonstrated a statistically significant reduction in ABG (*P* < 0.001).

Functional ABG closure (≤10 dB) was achieved in 70% of patients, while 25% had a residual ABG between 11 and 20 dB, and 5% exhibited an ABG greater than 20 dB postoperatively. Subjective hearing improvement was reported by 95% of patients. Postoperative audiological outcomes are summarized in Table 3.

Postoperative BC thresholds were systematically reviewed to assess cochlear safety. No clinically significant sensorineural deterioration, defined as ≥10 dB worsening in BC pure-tone average, was identified in this series.

High-resolution temporal bone CT was performed in all patients. Radiological evaluation, according to the Veillon classification, demonstrated a predominance of fenestral otosclerosis. Type 1B lesions were the most frequently observed (27.5%), followed by type 2 (25%) and type 1A (20%). Cochlear involvement (type 3) was identified in 17.5% of patients, while no cases of advanced labyrinthine otosclerosis (type 4) were observed. Radiological findings are summarized in Table 4.

All surgical procedures were successfully completed using a purely endoscopic transcanal approach. Intraoperative anatomical variations included an overhanging scutum (27.5%), a narrow external auditory canal (22.5%), and a prominent facial nerve (10%). Minor intraoperative incidents were limited to controlled bleeding (7.5%) and a floating footplate (5%). No major intraoperative complications occurred. Intraoperative findings are detailed in Table 5.

Postoperative complications were generally mild and transient. Transient vertigo occurred in 10% of patients and dysgeusia in 7.5%. No cases of facial nerve dysfunction, persistent vertigo, perilymphatic fistula, or delayed postoperative SNHL were observed during follow-up. Postoperative complications are detailed in Table 6.

## Discussion

Otosclerosis remains one of the leading causes of conductive hearing loss in adults with an intact tympanic membrane and continues to represent a major indication for stapes surgery^[^9^]^. Classical pathological and clinical descriptions have established the multifactorial nature of the disease, involving genetic predisposition, hormonal influences, and localized inflammatory processes^[^10^]^. In the present retrospective cohort, exclusive transcanal endoscopic stapedotomy resulted in a mean ABG improvement of 23 dB, with functional ABG closure (≤10 dB) achieved in 70% of patients and no clinically significant postoperative sensorineural deterioration. These findings support the feasibility, safety, and functional efficacy of the endoscopic approach when standardized surgical and audiological protocols are applied.

The demographic characteristics observed in our series are consistent with previously reported epidemiological patterns. Large clinical studies describe a peak incidence between the third and fifth decades of life, as reported by House and Cunningham and later confirmed by Declau *et al*^[^11^]^. The mean age of 34 years in our cohort aligns with these observations. Although otosclerosis has traditionally been reported to predominate in women, more recent surgical series – including those by Marchioni *et al* and Iannella *et al* – have demonstrated more balanced sex distributions^[^11^]^. This variability may reflect referral patterns or institutional case-mix rather than a true epidemiological shift.

Preoperative audiological findings in our study were consistent with the classical natural history of fenestral otosclerosis as described by Juers and Shambaugh^[^12^]^. The mean preoperative ABG of 32 ± 6 dB falls within the range commonly reported in both microscopic and endoscopic stapes surgery series, including those by Vincent *et al* and Kojima *et al*^[^12^]^. Importantly, postoperative ABG decreased significantly to 9 ± 4 dB (*P* < 0.001), confirming meaningful functional improvement. The absence of BC deterioration ≥10 dB in our cohort indicates preservation of cochlear function, which remains a critical safety endpoint in stapes surgery. Reported rates of postoperative SNHL in the literature range between 0 and 2%, and our findings align with these safety benchmarks^[^13,14^]^.

High-resolution CT of the temporal bone plays a central role in confirming diagnosis and optimizing surgical planning^[^15^]^. The Veillon classification provides a standardized radiological framework for assessing fenestral and cochlear involvement. In our series, fenestral otosclerosis (types 1A, 1B, and 2) predominated, consistent with reports by Purohit and Hermans and Rotteveel *et al*^[^15^]^. The inclusion of Veillon Type 0 cases warrants clarification: high-resolution CT may be normal in early fenestral otosclerosis, and intraoperative confirmation of stapes fixation remains the diagnostic gold standard. Therefore, the inclusion of CT-negative cases reflects clinical reality rather than diagnostic uncertainty. The absence of advanced labyrinthine involvement (type 4) likely contributed to the favorable postoperative audiological outcomes observed.

From a technical standpoint, all procedures were performed as exclusive endoscopic stapedotomy using a 0.6-mm Teflon piston under general anesthesia. The feasibility and safety observed in our series are in accordance with the pioneering work of Tarabichi, who first highlighted the advantages of endoscopic visualization in middle ear surgery^[^16^]^. Subsequent studies by Marchioni *et al*, Presutti *et al*, and Kojima *et al* demonstrated that the endoscopic approach provides a wide-angle, high-definition view of the stapes suprastructure, oval window niche, facial nerve, and ossicular chain^[^16^]^. In our cohort, anatomical challenges such as overhanging scutum or narrow external auditory canal did not compromise surgical exposure, supporting observations reported by Iannella *et al* and Tseng *et al*^[^16^]^. These findings reinforce the reproducibility of the endoscopic approach when performed by experienced surgeons.

Functionally, our ABG closure rate of 70% is comparable to outcomes reported in both endoscopic and microscopic techniques, where closure ≤10 dB ranges from approximately 60–90%^[^17^]^. Several comparative studies and systematic reviews have concluded that endoscopic and microscopic stapes surgery yield equivalent hearing results^[^17,18^]^. These data suggest that the endoscopic approach does not compromise functional efficacy while offering improved visualization of the middle ear anatomy.

The complication profile in our study was favorable. Postoperative events were limited to transient vertigo and dysgeusia, with no cases of facial nerve dysfunction or perilymphatic fistula. Similar findings have been reported by Vincent *et al*, Sennaroglu and Gursel, and more recently by Iannella *et al*, emphasizing that complication rates are closely related to surgical expertise rather than the choice of optical instrument^[^13,14^]^. These results further support the safety of exclusive endoscopic stapedotomy when performed by surgeons experienced in endoscopic otologic techniques.

Several limitations should be acknowledged. The retrospective design introduces inherent selection bias, and the absence of a control group undergoing microscopic stapedotomy precludes direct comparative conclusions. The sample size remains modest, and follow-up was limited to short-term postoperative assessment. Long-term audiological stability and prosthesis durability were not evaluated. Nevertheless, as emphasized by Presutti *et al* and Marchioni *et al*, accumulating evidence from multiple centers supports the role of endoscopic stapes surgery as a reliable and reproducible technique^[^19^]^.

In summary, in agreement with previous reports by Tarabichi, Marchioni, Iannella, and Gioacchini, our findings confirm that exclusive transcanal endoscopic stapedotomy is a safe and effective option for the surgical management of otosclerosis^[^8,16,17,19^]^. With appropriate patient selection and sufficient surgical expertise, the endoscopic approach provides excellent visualization, significant hearing improvement, and low morbidity, supporting its integration into contemporary otologic surgery.

## Conclusion

Endoscopic transcanal stapedotomy represents a safe, effective, and reproducible surgical technique for the management of otosclerosis. In this retrospective cohort, the exclusive endoscopic approach provided enhanced visualization of middle ear anatomy and enabled precise prosthesis placement, resulting in a significant mean ABG improvement of 23 dB (*P* < 0.001), with functional closure (≤10 dB) achieved in 70% of patients. No clinically significant postoperative sensorineural deterioration was observed, supporting the cochlear safety of the technique. These findings align with contemporary literature and reinforce the role of endoscopic stapes surgery as a reliable alternative to conventional microscopic approaches in appropriately selected patients.

## Data Availability

The datasets generated and/or analyzed during the current study are available from the corresponding author upon reasonable request.
